# Single-Piece Solid Contact Cu^2+^-Selective Electrodes Based on a Synthesized Macrocyclic Calix[4]arene Derivative as a Neutral Carrier Ionophore

**DOI:** 10.3390/molecules24050920

**Published:** 2019-03-06

**Authors:** Abd El-Galil E. Amr, Mohamed A. Al-Omar, Ayman H. Kamel, Elsayed A. Elsayed

**Affiliations:** 1 Pharmaceutical Chemistry Department, Drug Exploration & Development Chair (DEDC), College of Pharmacy, King Saud University, Riyadh 11451, Saudi Arabia; malomar1@ksu.edu.sa; 2 Applied Organic Chemistry Department, National Research Centre, Dokki, Cairo 12622, Egypt; 3 Chemistry Department, Faculty of Science, Ain Shams University, Abbasia, Cairo 11566, Egypt; 4 Zoology Department, Bioproducts Research Chair, Faculty of Science, King Saud University, Riyadh 11451, Saudi Arabia; eaelsayed@ksu.edu.sa; 5 Chemistry of Natural and Microbial Products Department, National Research Centre, Dokki, Cairo 12622, Egypt

**Keywords:** solid-contact, ion-selective electrode, single-walled carbon nanotubes (SWCNTs), PEDOT/PSS, copper

## Abstract

Herein, a facile route leading to good single-walled carbon nanotubes (SWCNT) dispersion or poly (3,4-ethylenedioxythiophene)/poly(styrenesulfonate) (PEDOT/PSS) based single-piece nanocomposite membrane is proposed for trace determination of Cu^2+^ ions. The single-piece solid contact Cu^2+^-selective electrodes were prepared after drop casting the membrane mixture on the glassy-carbon substrates. The prepared potentiometric sensors revealed a Nernstian response slope of 27.8 ± 0.3 and 28.1 ± 0.4 mV/decade over the linearity range 1.0 × 10^−3^ to 2.0 × 10^−9^ and 1.0 × 10^−3^ to 1.0 × 10^−9^ M with detection limits of 5.4 × 10^−10^ and 5.0 × 10^−10^ M for sensors based on SWCNTs and PEDOT/PSS, respectively. Excellent long-term potential stability and high hydrophobicity of the nanocomposite membrane are recorded for the prepared sensors due to the inherent high capacitance of SWCNT used as a solid contact material. The sensors exhibited high selectivity for Cu^2+^ ions at pH 4.5 over other common ions. The sensors were applied for Cu^2+^ assessment in tap water and different tea samples. The proposed sensors were robust, reliable and considered as appealing sensors for copper (II) detection in different complex matrices.

## 1. Introduction

It is important for environmental protection, food and agricultural chemistry to monitor and quantify metal ions in the trace level. Copper is widely used in industry, agriculture and for domestic purposes. Therefore, it can be considered as one of the most widely distributed elements in industrialized countries. Copper has a major role in numerous biological processes such as blood formation and the functioning of various enzymes [1,2]. The maximum tolerable level for copper is 2.0 mg/L, and an excessive intake of this element manifests certain diseases in humans such as Menkes syndrome and Wilson’s disease [3,4]. Consequently, it is necessary to find an accurate and reliable analytical method for trace-level determination of copper. This has become imperative from the viewpoint of its utility as well as its toxicity.

Several analytical techniques have been reported for copper determination [5,6,7,8,9,10,11,12,13,14,15,16,17,18]. Among these, Cu^2+^-selective electrodes integrated in potentiometric systems, especially the solid contact ion-selective electrodes (SC-ISEs), draw a lot of attraction owing to their portability, disposability and low-cost [19,20,21,22]. These potentiometric type sensors have wide applications in many fields, such as medical diagnosis, process control and environmental monitoring [23,24,25]. Coated wire electrode (CWE) is the original part of SC-ISE in which the ion-selective membrane (ISM) is directly coated on a conductive substrate [26]. CWEs have limited applications due to their poor reproducibility and potential instability. These are caused by the blocked (low capacity) interface between the electronic conductor and the ionically conducting ISM [27]. This ill-defined interface between the ISM and the conducting substrate leads to potential drift. Therefore, much work has focused on developing ion-to-electron transducing layers to form an ion exchange equilibrium-based stable potential at the ISM/SC interface and phase boundary potential at the SC/electronic conductor interface. This work includes conducting polymers (CPs) [28] and nano-structured carbon materials [29,30,31,32].

Several reports focus on using solid contact materials-based solid-contact Cu^2+^-ISE. One is based on the use of 1-ethyl-3-methyl imidazolium chloride as an ionic liquid doped with the ionophore [33]. Nano-structured carbon materials are also used as solid contact materials-based solid-contact Cu^2+^-ISE such as carboxylic multi-walled carbon nanotubes [34], multi-walled carbon nanotubes (MWCNT) [35], single-walled carbon nanotubes (MWCNT) [36] and graphen (GR) and grapheme oxide (GO) [37]. Single-piece solid-contact Cu^2+^-ISEs have different advantages such as ease of miniaturization, simplicity, stability and disposability.

In this work, a facile approach for the fabrication of single-piece Cu^2+^-selective nanocomposite membrane based on two different solid contact materials, namely surfactant-free and well-dispersed single walled-carbon nanotube (SWCNT) and poly (3,4-ethylenedioxythiophene)/poly(styrenesulfonate) (PEDOT/PSS) are proposed here. The potentiometric performance characteristics of the single-piece Cu^2+^-ISE nanocomposite membrane were studied as well as the sensing conditions. The practical feasibility of the proposed sensors was evaluated in copper determination in tap water and different tea samples collected from the local market.

## 2. Results and Discussion

### 2.1. SWCNTs Versus PEDOT/PSS

**Figure 1:**
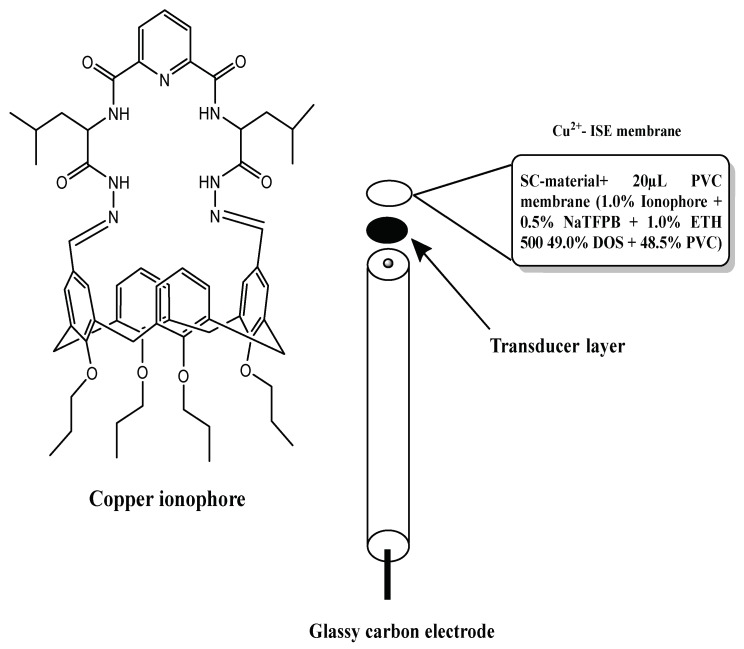
Copper ionophore and electrode design.

In this work, we employed a facile and simple method for fabrication of robust solid-contact ISEs using a synthesized macrocyclic calix[4]arene derivative as copper Ionophore (Figure 1) and SWCNTs and PEDOT/PSS as solid-contact transducers. The single-piece solid contact Cu^2+^-ISEs were prepared via one-step drop casting the ultra-sonicated suspension mixture on GC surface electrodes. The principle of ion-to-electron transduction in SWCNTs is based on the high charge transfer of carbon nanotubes. SWCNTs are characterized by their high surface/volume ratios. This contributes to enlarging the contact area between the polymeric sensing membrane and the nanotubes. As such, it will minimize the resistance at this interface. The high influence of the surrounding chemical environment on the conductance of carbon nanotubes makes the nanotubes very sensitive to the phase-boundary potential changes generated at the interface ion-selective membrane-test solution. The charge transfer process between the ionic conducting membrane and the electronically conducting wire takes place without producing voltage instabilities. SWCNTs are also characterized by their high hydrophobicity. This may be the reason why a significant water layer is not detected in the ion-selective membrane or the transducer layer interface.

Poly (3,4-ethylenedioxythiophene) is known as one of the most stable conducting polymers available today [38]. The principle of ion-to-electron transduction in PEDOT as a solid-contact material is directly related to the redox capacitance of this material. PEDOT as a conducting polymer is characterized by high stability, high ionic mobility and a high charge transfer rate, as well as good adhesion to the membrane material and electrode substrate. These characteristics make it a good material for a solid contact of ISEs.

**Figure 2:**
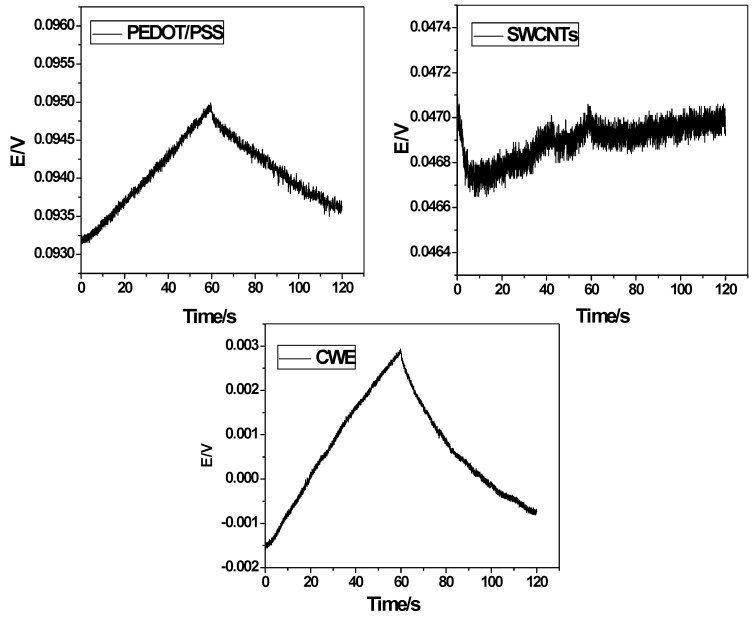
Chronopotentiometric results done for GC/Cu^2+^-ISEs (CWE), GC/PEDOT/PSS/Cu^2+^-ISEs and GC/SWCNTs/Cu^2+^-ISEs, applying anodic/cathodic current of 10 nA, in 0.01 M NaCl.

To evaluate the potential stability in the proposed sensors using either PEDOT/PSS or SWCNTs as solid contact materials, constant current chronopotentiometry was used according to Bobacka’s protocol [39]. As can be seen from the chronopotentiograms shown in Figure 2, the bulk membrane resistances together with contact resistance between the ISM and the underlying conductor (*R_bc_*) were found to be 0.2 ± 0.03, 0.11 ± 0.04 and 0.10 ± 0.02 MΩ, for GC/Cu^2+^, GC/PEDOT/PSS/Cu^2+^ and GC/SWCNTs/Cu^2+^-ISEs, respectively. For GC/Cu^2+^-ISEs (i.e., CWE), the potential drift was calculated to be 75 ± 3.2 µV s^−1^. Nevertheless, GC/PEDOT/PSS/Cu^2+^ and GC/SWCNTs/Cu^2+^-ISEs a negligible potential drift was observed, and a much smaller potential drift value of 30.1 ± 2.5 µV s^−1^ (*n* = 3) and 16.6 ± 1.2 µV s^−1^ (*n* = 3) was recorded, respectively. As mentioned, the potential stability of SWCNTs is much higher than of PEDOT/PSS. This is confirmed by the measured capacitance for both. The capacitances of GC/Cu^2+^, GC/PEDOT/PSS/Cu^2+^ and GC/SWCNTs/Cu^2+^-ISEs were calculated to be 13.3 ± 0.7, 33.3 ± 1.3 and 60.2 ± 0.2 µF, respectively.

### 2.2. Response Characteristics of the Solid-Contact Cu^2+^-ISEs

**Figure 3:**
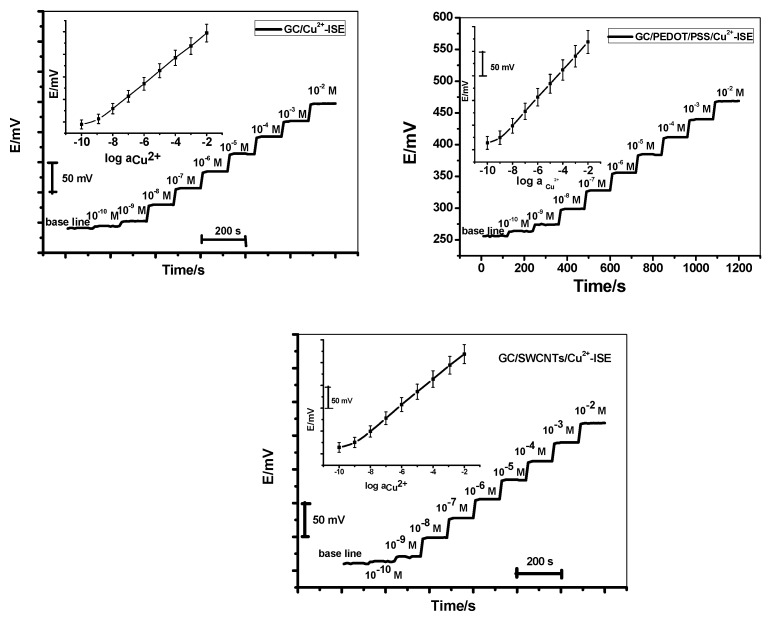
Recorded potential time traces for the proposed Cu^2+^-ISEs. The inset shows the corresponding calibration curves.

Five different sensor assemblies for each membrane type were tested over a period of 3 months. All potentiometric characteristics were shown in Table 1. The potentiometric response of the sensors at pH 4 revealed Nernstian slopes of 28.0 ± 0.4, 28.1 ± 0.4, and 27.8 ± 0.3 mV/decade over the linear concentration range of 1.0 × 10^−9^ to 1.0 × 10^−3^, 2.0 × 10^−9^ to 1.0 × 10^−3^ and 1.0 × 10^−9^ to 1.0 × 10^−3^ M with a detection limits 5.0 × 10^−10^, 5.4 × 10^−10^ and 5.0 × 10^−10^ for GC/Cu^2+^, GC/PEDOT/PSS/Cu^2+^ and GC/SWCNTs/Cu^2+^- ISEs, respectively (Figure 3).

The dependence of electrode potential on pH was investigated using 1 × 10^−4^ M Cu^2+^ solution over the pH range 2.0–7.0. The pH was adjusted using HNO_3_ or LiOH solutions. According to the obtained results, the potential of proposed sensors is almost constant over the pH range 3–6.5 as shown in Table 1. At pH < 6.5, the potential declined due to the formation of Cu(OH)_2_. At pH < 3.0, the potential begins to increase due to the acidic media effect, which may be attributed to either the ionic additive in the membrane phase or the ionophore properties or both.

Sensor selectivity is one of the most important parameters for any potentiometric sensor. Selectivity coefficient values were calculated using the separate solution method (SSM) to eliminate the influence of the inherent sensitivity limit on the ISE response toward the discriminated ions [40]. As shown in Table 2, the sensors having SWCNTs in the membrane are characterized by better selectivity than those containing PEDOT/PSS. This may be attributed to the contribution of ionic exchange property of both [PEDOT]^+^ and [PSS]^−^ ions.

Electrode potential reproducibility was also investigated. Six electrodes from each membrane type were taken and the measurements were conducted in 10^−2^ M Cu^2+^ solution with pH adjusted to 4.0 with HNO_3_ as the background. The mean value of the slope for each membrane type sensor is 28.2 ± 0.7, 27.8 ± 0.3 and 28.1 ± 0.4 mV/decade, and the standard deviation is 1.3, 0.7 and 0.8 mM for GC/Cu^2+^, GC/PEDOT/PSS/Cu^2+^ and GC/SWCNTs/Cu^2+^-ISEs, respectively.

### 2.3. Water Film Test of the Electrode Potential

**Figure 4:**
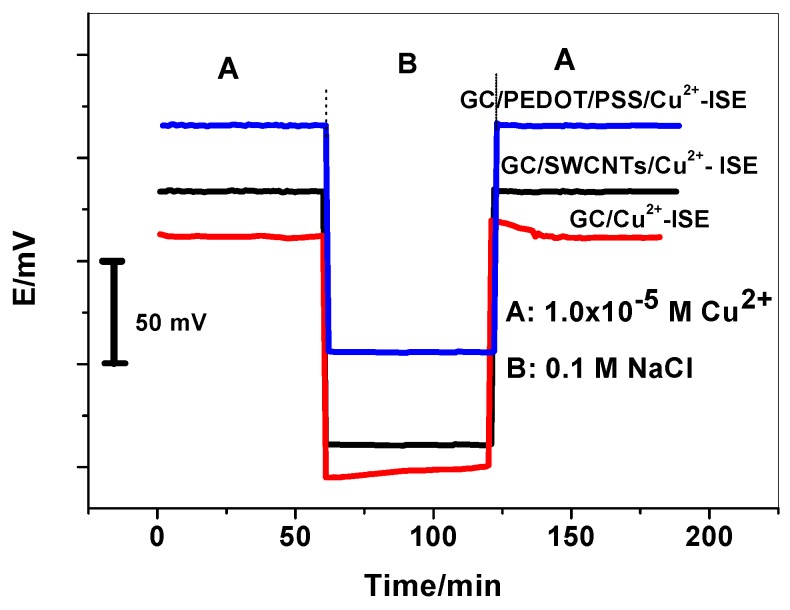
Water film tests of the solid-contact Cu^2+^-ISEs and of the CWE. At t *=* 1 h, the solution of the primary ion (10^−5^ M Cu^2+^) was exchanged to 10^−1^ M NaCl. At t *=* 2 h, the initial solution was inserted.

Formation of water film between the Cu^2+^-selective membrane and the solid contact material was investigated. In this test, the ISEs were first conditioned in a solution of the primary ions and then the sample was replaced with a solution of the background electrolyte. A control experiment was performed by using the CWE. As shown in Figure 4, a negative EMF change of ~115 mV is noticed for all ISEs upon replacing 1.0 × 10^−5^ M Cu^2+^ solution with the electrolytic background solution. After 3 h, a positive potential drift is recorded for the CWE indicating the formation of water layer between the electrode membranes and conducting substrate. For sensors based on PEDOT and SWCNTs as solid contact materials, they exhibit a stable potential response and confirm the absence of a water layer. From these results, we can demonstrate that the undesirable water layer can be successfully eliminated by the intermediate solid contact layer of the proposed sensors. This is can be attributed to the high hydrophobicity of the PEDOT and SWCNTs based transducer layer, which may lead to the absence of the water film.

The long-term response of the proposed GC/SWCNTsCu^2+^ and GC/PEDOT/PSS/Cu^2+^-ISEs is also tested. When not in use, these fabricated sensors were all conditioned in 1.0 × 10^−9^ M Cu^2+^ solution. No noticeable change in the response slopes and detection limits was observed within 3 months. After three months, the detection limit changed about half an order of magnitude. The results indicated the absence of the water films. The robust and reliable single-piece Cu^2+^-ISEs are promising for applications in many fields of contemporary research.

### 2.4. Sensors’ Applicability

To test the applicability of the sensors, they were introduced to assess copper content in collected tap water samples and tea samples collected from the local market. As shown in Table 3, it can be seen that the recoveries of tap water samples vary from 93.3% to 106.6% and the data obtained by the proposed electrode agree well with those obtained by the AAS method. This confirms that the proposed potentiometric sensor has a promising potential for real sample analysis and feasible for assessing Cu^2+^ ions in polluted water samples.

The sensor was also used for the direct assay of copper content in tea infusion samples collected from the local market. The obtained results were compared with AAS method for the evaluation of the accuracy. All measurements were carried out in triplicate. The results obtained are shown in Table 4. The data obtained showed that potentiometric measurements of Cu^2+^ ions in tea samples were in good agreement with those obtained by the AAS method. For the statistical treatment of data; Student *t* test was performed at 95% confidence level. For all of the tea samples the calculated t values at 95% confidence levels are smaller than the critical t value (2.78). According to this, no significant differences between the performances of the two methods and the proposed sensor. The proposed sensors show a good applicability in the assessment of Copper content in real samples.

To explore the novelty of this work, comparison of the proposed sensors with other solid-contact Cu^2+^-ISEs previously reported is also given in Table 5. The table clearly indicated good enhancement in the behavior of the proposed copper electrode in terms of the detection limit and linear range [33,35,36,37,41,42,43,44], slope [34,44] and selectivity [33,34,42].

## 3. Materials and Methods

### 3.1. Reagents

Copper ionophore (Figure 1) was prepared and elucidated according to literature procedures [45]. High molecular weight poly(vinyl chloride) (PVC), tetradodecylammonium tetrakis (4-chlorophenyl) borate (ETH 500), sodium tetrakis [3,5 *bis* (trifluoromethyl) phenyl] borate (NaTFPB), 2-nitrophenyl octyl ether (*o*-NPOE) and tetrahydrofuran (THF) were all purchased from Sigma-Aldrich (Gillingham, Dorset, UK). SWCNTs obtained from XFnano Materials Tech Co., Ltd. (Nanjing, China) with no further purification and modification. Copper nitrate (Cu(NO_3_)_2_) and other salts were all obtained from Sigma-Aldrich, and corresponding solutions were prepared in freshly deionized water (DI water, resistance 18.2 MΩ cm, Millipore, Washington, WA, USA). Other chemicals used in this study were all of analytical reagent grade and used as received.

A stock solution of 0.01 M Cu^2+^ was prepared by dissolving Cu(NO_3_)_2_ in deionized water and then diluted to various concentrations of working solutions with deionized water prior to measurements.

### 3.2. Apparatus

All measurements of electromotive force (EMF) were performed at ambient temperature using an Orion pH/mV meter (model SA 720, Cambridge, MA, USA) in the galvanic cell: Ag/AgCl/KCl (sat)//0.1 M LiOAc/sample solution/ISE membrane containing SC material/GCE. Selectivity coefficients were determined by the separate solution method [39]. Chronopotentiometry tests were done using a conventional three electrode system including an ISE working electrode, Ag/AgCl (3 M) as the reference electrode and a Pt wire as the counter electrode. Chronopotentiometric measurements were carried out on the proposed sensors in 10^−2^ M Cu^2+^ by applying a constant current of ±1 nA for 60 s, respectively.

### 3.3. Electrode Fabrication

The ion-selective sensing nanocomposite membrane cocktail (total mass 360 mg in 2.5 mL THF) was prepared by dissolving copper ionophore (1 wt%,), NaTFPB (0.5 wt%), ETH 500 (1 wt%), *o*-NPOE (49 wt%), and PVC (48.5 wt%) together with 1 mg of SWCNT in 1 mL THF or 20 µL of PEDOT/PSS solution. The cocktail was sonicated for at least 30 min with a power of 200 W to obtain a uniform solution. The sensors were fabricated as follows: Glassy carbon electrodes (GCE) were polished with 0.3 µm alumina slurries, sonicated with ethanol and de-ionized water separately, then dried under nitrogen. The cleaned electrodes were then tightly inserted into a piece of matched PVC tube (1 cm long, 5 mm i.d. and 8 mm o.d.) at the distal end.

A 200 μL of the membrane cocktail was dropped cast on the GC disks evenly and the solvent was evaporated thoroughly at ambient temperature. A uniform composite layer with strong adhesion to the GCE surface was obtained. The coated wire electrodes (CWEs) were prepared by the similar steps without the addition of solid contacts. The Cu^2+^-selective electrodes were firstly conditioned in 10^−3^ M Cu^2+^ ion solution for 1 day and then in 10^−9^ M Cu^2+^ for 2 days. The pH of the test solutions was adjusted by HNO_3_ at pH 4. This pH of the tested copper nitrate solution makes Cu^2+^ the predominating form of copper.

### 3.4. Analytical Application

The sensors were introduced to determine copper content in different samples. Different tap water samples were collected and analyzed by the proposed GC/SWCNT Cu^2+^-ISEs to analyze the Cu^2+^ concentration. The samples were first boiled for about 10 min to remove excess chlorine present and adjusted with HNO_3_ till the pH of the sample reaches 3.8. A comparison between the proposed sensor and the atomic absorption spectroscopy (AAS) method was performed.

The sensors were also used for the direct assay of copper content in tea infusion samples collected from the local market. About a 100 gm portion of the tea samples were introduced into a porcelain dish and ashed at 300 °C for 1 h then heated in a muffle furnace at 1000 °C for an extra 2 h. After cooling, 2 mL of concentrated HNO_3_ and 2 mL of concentrated HCl were added and the content was heated until near dryness. The residue was dissolved by deionized water and completed to the mark in 50 mL measuring flask. A 10.0-mL aliquot of the filtrate was transferred into a 30-mL beaker and the copper content was determined using the proposed potetiometric method.

## 4. Conclusions

In this work, an all-solid-state potentiometric sensor for determination of copper has been proposed. It is based on the synthesized macrocyclic calix[4]arene derivative as the selective receptor and the SWCNTs and PEDOT/PSS as the solid contact materials on glassy carbon substrate. The solid-contact material was dispersed into *o*-NPOE-plasticized membrane by ultrasonication. The simple and generally applicable one-step fabrication yielded a single piece Cu^2+^-ISEs. The sensors revealed low detection limit of 5.4 × 10^−10^ and 5.0 × 10^−10^ M and a fast response time of <10 s for GC/SWCNTs-Cu^2+^-ISEs and GC/PEDOT/PSS/Cu^2+^-ISEs, respectively. The addition of either SWCNTs or PEDOT/PSS into the Cu^2+^-selective membrane enhanced the hydrophobicity and capacitance with considerable potential stability which was tested by electrochemical impedance spectroscopy (EIS) and constant-current chronopotentiometry techniques. In addition, a water layer test was also conducted to confirm the absence of water films between the ion-selective membrane and the electron conductor. The proposed single-piece Cu^2+^-ISEs were successfully applied for copper quantification in both tea and tap water samples. The proposed sensors offered good advantages such as simplicity in fabrication, easy operation, robustness, stability and low cost.

## Figures and Tables

**Table 1:** Response characteristics of solid contact-Cu^2+^-ISEs.

Parameter	GC/Cu^2+^-ISE (CWE)	GC/SWCNTs/Cu^2+^-ISE	GC/PEDOT/PSS/Cu^2+^-ISE
Slope (mV/decade)	28.0 ± 0.4	27.8 ± 0.3	28.1 ± 0.4
Correlation coefficient (r^2^)	0.9993	0.9997	0.9990
Detection limit (M)	5.0 × 10^−10^	5.4 × 10^−10^	5.0 × 10^−10^
Linear range (M)	1.0 × 10^−9^–1.0 × 10^−2^	2.0 × 10^−9^–1.0 × 10^−2^	1.0 × 10^−9^–1.0 × 10^−2^
Response time (s)	<5	<5	<5
Working pH range (pH)	3.0–6.5	3.0–6.5	3.0–6.5
Accuracy (mV%)	99.6	99.3	98.8
Precision (mV%)	1.1	1.2	1.7
Between-day variability (mV%)	0.9	1.5	1.3

**Table 2:** Potentiometric selectivity coefficients (*log K_Cu, J_*) ± SD obtained for the proposed sensors.

Interfering Ion, *J*	*log K_Cu, J_* ± SD		
	GC/Cu^2+^-ISE	GC/SWCNTs-Cu^2+^-ISE	GC/PEDOT/PSS/Cu^2+^-ISE
Na^+^	−6.5 ± 0.3	−6.6 ± 0.4	−6.3 ± 0.3
Mg^2+^	−8.4 ± 0.1	−8.5 ± 0.3	−8.4 ± 0.4
Ca^2+^	−8.8 ± 0.2	−8.7 ± 0.1	−8.6 ± 0.3
K^+^	−5.1 ± 0.7	−5.0 ± 0.6	−5.0 ± 0.5
Pb^2+^	−4.5 ± 0.8	−4.3 ± 0.4	−4.4 ± 0.1
Cd^2+^	−7.6 ± 0.2	−7.6 ± 0.3	−7.4 ± 0.4
Zn^2+^	−7.2 ± 0.3	−7.3 ± 0.1	−7.4 ± 0.2
Ni^2+^	−8.1 ± 0.7	−8.2 ± 0.4	−8.0 ± 0.3
Ag^+^	−3.9 ± 0.4	−3.7 ± 0.7	−3.8 ± 0.4
Hg^2+^	−4.1 ± 0.5	−4.2 ± 0.3	−4.1 ± 0.4

**Table 3:** Application of the proposed sensor to determination of Cu^2+^ ions in tap water samples.

Sample	(µg/mL) *				Recovery (%)
	Proposed Sensor	AAS	Added	Found	
Sample 1	1.10 ± 0.2	1.05 ± 0.3	0.3	1.42 ± 0.1	106.6
			0.5	1.62 ± 0.2	104.0
			0.8	1.87 ± 0.3	96.2
Sample 2	0.80 ± 0.07	0.82 ± 0.04	0.3	1.08 ± 0.4	93.3
			0.5	1.31 ± 0.2	102.0
			0.8	1.58 ± 0.1	97.5

* Average of 5 measurements.

**Table 4:** Copper assessments in tea samples using solid contact Cu^2+^-ISE.

Tea Samples	Copper Content ± SD (mg/Kg) ^a^		*t*-Test ^b^
	Potentiometry	AAS	
Lipton (Black Sri Lankan Tea, Cairo, Egypt)	8.3 ± 0.7	8.0 ± 0.3	2.37
Ahmed Tea (Black Tea, London, UK)	12.3 ± 0.5	12.6 ± 0.4	2.61
Al-Arosa (Dust Black Kenyan tea, Cairo, Egypt)	23.4 ± 0.8	22.8 ± 0.2	2.53
Al-Rabea (Black Tea, Riyadh, Saudi Arabia)	21.3 ± 0.6	22.1 ± 0.3	2.67
Dilmah (Sri Lankan Tea, London, UK)	17.3 ± 0.8	16.7 ± 0.1	2.43

^a^ Average of three replicate measurements ± standard deviation. ^b^ The theoretical values of t at *p* = 0.05 is 2.78.

**Table 5:** Comparison of the potentiometric characteristic response of the presented ISEs and other previously reported solid contact Cu^2+^-ISEs.

Ionophore	Solid Contact Material	Slope, mV/Decade	Detection Limit, M	Linear Range, M	Potential Drift, µV/s	Capacitance, µF	Selectivity Coefficients, *log K_Cu_^2+^_, J,_* Method Used	Ref.
*o*-Xylylene *bis* (*N*,*N*-diisobutyldithiocarbamate	Graphite	31.3	4.9 × 10^−7^	1.0 × 10^−6^–1.0 × 10^−2^	NR	NR	Co^2+^ (−3.8), Na^+^ (−4.7), K^+^ (−2.4), Zn^2+^ (−5.2), Ba^2+^ (−4.5), NH_4_^+^ (−4.1), Ni^2+^ (−2.3), Cd^2+^ (−3.0), Ca^2+^ (−3.5), Pb^2+^ (−2.5). SSM	[44]
*N*,*N*,*N*′,*N*′-Tetradodecyl-3,6-dioxaoctanedithioamide	SWCNTs	29.8	4.0 × 10^−9^	1.0 × 10^−4^–1.0 × 10^−8^	5.2		Na^+^ (−10.5), K^+^ (−8.6), Ca^2+^ (−11.9), Mg^2+^ (−13.3). SSM	[36]
*N,N,N′,N′*-Tetracyclohexyl-2,2′-thiodiacetamide	1-Ethyl-3-methyl imidazolium chloride.	28.9	3.2 × 10^−8^	1.0 × 10^−7^–1.0 × 10^−1^	NR	NR	Co^2+^ (−3.16), Na^+^ (−4.95), K^+^ (−5.21), Zn^2+^ (−3.39), Mg^2+^ (−6.22), Li^+^ (−5.11), Ni^2+^ (−3.02), Cd^2+^ (−3.84), Ca^2+^ (−4.93). SSM	[33]
1-(2-Aminoethyl)-3-butyl imidazolium *bis* (trifluoromethane sulfonyl) imide	Carboxylic multi-walled carbon nanotubes (MWCNTs-COOH)	8.17	7.9 × 10^−11^	1.0 × 10^-10^–1.0 × 10^−5^	NR	NR	Co^2+^ (−2.7), Na^+^ (−4.1), K^+^ (−3.9), Zn^2+^ (−2.5), Mg^2+^ (−3.4), NH_4_^+^ (−3.5), Ni^2+^ (−3.0), Mn^2+^ (−3.7), Ca^2+^ (−3.0), Pb^2+^ (−3.1), Cr^3+^ (−3.3), Fe^3+^ (−3.3). FIM	[34]
(*bis*-[(2-(Hydroxyethylimino) phenolato]copper(II))	NR	28.3	8.3 × 10^−7^	1.0 × 10^−6^−1.0 × 10^−1^	NR	NR	Na^+^ (−4.3), K^+^ (−4.3), Ca^2+^ (−4.4), Ba^2+^ (−4.5), Pb^2+^ (−3.8), Zn^2+^ (−3.0), Co^2+^ (−3.1), Ni^2+^ (−3.2), Cd^2+^ (−4.6), Cr^3+^ (−1.6). SSM	[42]
3-(2-Methyl-2,3-dihydrobenzothiazol-2-yl)-2*H*-chromen-2-one	MWCNTs	29.3	7.9 × 10^−7^	1.0 × 10^−6^−1.0 × 10^−1^	NR	NR	Hg^2+^ (−2.3), Lu^3+^ (−2.5), K^+^ (−3.1), Zn^2+^ (−3.9), Gd^2+^ (−3.8), Ag^+^ (−3.7), Ni^2+^ (−3.8), Mn^2+^ (−2.8), Cd^2+^ (−3.8), Ca^2+^ (−3.1), Pb^2+^ (−3.5), Cr^3+^ (−2.3), Fe^3+^ (−2.8), La^3+^ (−3.2). MPM	[35]
7,7,8,8-Tetracyanoquinodimethane	Graphen (GR) Graphenoxide (GO)	30.5 30.6	1.0 × 10^−9.2^ 1.0 × 10^−7.5^	1.0 × 10^−9^–1.0 × 10^−2^ 1.0 × 10^−7^–1.0 × 10^−2^	20.2 54.5	495 183	K^+^ (−5.02), Na^+^ (−5.26), Ag^+^ (3.40), Mg^2+^ (−5.89), Ca^2+^ (−5.06), Zn^2+^ (−2.49), Pb^2+^ (−1.88), Ni^2+^ (−2.52). SSM K^+^ (−5.52), Na^+^ (−6.27), Ag^+^ (3.42), Mg^2+^ (−6.84), Ca^2+^ (−5.76), Zn^2+^ (−2.94), Pb^2+^ (−2.76), Ni^2+^ (−2.97). SSM	[37]
Dithizone	Gold nanoparticle	23.5 24.1 22.1	1.0 × 10^−5.5^ 1.0 × 10^−6^ 1.0 × 10^−7.5^	1.0 × 10^−5^–1.0 × 10^−1^ 1.0 × 10^−5^–1.0 × 10^−1^ 1.0 × 10^−7^–1.0 × 10^−1^	NR NR NR	11 14 13	K^+^ (−3.8), Na^+^ (−6.2), Mg^2+^ (−8.2), Ca^2+^ (−8.7), Zn^2+^ (−7.1), Pb^2+^ (−3.2), Ni^2+^ (−8.1), Cd^2+^ (−6.0). SSM K^+^ (−3.0), Na^+^ (−5.2), Mg^2+^ (−7.3), Ca^2+^ (−7.8), Zn^2+^ (−6.3), Pb^2+^ (−3.1), Ni^2+^ (−7.2), Cd^2+^ (−5.4). SSM K^+^ (−2.3), Na^+^ (−2.9), Mg^2+^ (−4.4), Ca^2+^ (−6.1), Zn^2+^ (−4.1), Pb^2+^ (−1.7), Ni^2+^ (−4.5), Cd^2+^ (−4.5). SSM	[43]
1,2-di-(*o*-Salicylaldimino -phenylthio) ethane	Carbon ink	31.0	1.6 × 10^−^^6^	3.2 × 10^−^^6^–2.8 × 10^−^^2^	NR	NR	K^+^ (−7.0), Na^+^ (−7.0), Mn^2+^ (−2.6), Ca^2+^ (−7.0), Zn^2+^ (−2.3), Pb^2+^ (−2.0), Ni^2+^ (−7.0), Cd^2+^ (−4.1), Ag^+^ (−7.0), Co^2+^ (−5.0), Fe^2+^ (−5.0). MPM	[41]
Macrocyclic calix[4]arene derivative	SWCNTs PEDOT/PSS	27.8 ± 0.3 28.1 ± 0.4	5.4 × 10^−10^ 5.0 × 10^−10^	1.0 × 10^−3^–2.0 × 10^−9^ 1.0 × 10^−3^–1.0 × 10^−9^	30.1 ± 2.5 16.6 ± 1.2	33.3 ± 1.3 60.2 ± 0.2	Mg^2+^ (−8.5), Na^+^ (−6.6), K^+^ (−5.0), Zn^2+^ (−7.3), Hg^2+^ (−4.2), Ag^+^ (−3.7), Ni^2+^ (−8.2), Cd^2+^ (−7.6), Ca^2+^ (−8.7), Pb^2+^ (−4.3). SSM Mg^2+^ (−8.4), Na^+^ (−6.3), K^+^ (−5.0), Zn^2+^ (−7.4), Hg^2+^ (−4.1), Ag^+^ (−3.8), Ni^2+^ (−8.0), Cd^2+^ (−7.4), Ca^2+^ (−8.6), Pb^2+^ (−4.4). SSM	This work

SSM: Separate solution method. MPM: Matched potential method. FIM: Fixed interference method. NR: Not reported.
